# Neuroglia Cells Transcriptomic in Brain Development, Aging and Neurodegenerative Diseases

**DOI:** 10.14336/AD.2022.0621

**Published:** 2023-02-01

**Authors:** Leonard Radu Pinosanu, Bogdan Capitanescu, Daniela Glavan, Sanziana Godeanu, Israel Ferna´ndez Cadenas, Thorsten R. Doeppner, Dirk M. Hermann, Adrian-Tudor Balseanu, Catalin Bogdan, Aurel Popa-Wagner

**Affiliations:** ^1^Experimental Research Center for Normal and Pathological Aging (ARES), University of Medicine and Pharmacy of Craiova, Craiova, Romania.; ^2^Psychiatric clinic, University of Medicine and Pharmacy Craiova, Craiova, Romania.; ^3^Stroke Pharmacogenomics and Genetics group, Sant Pau Hospital Institute of Research, Barcelona, Spain.; ^4^Department of Neurology, University Hospital Giessen, Giessen, Germany.; ^5^University of Göttingen Medical School, Department of Neurology, Göttingen, Germany.; ^6^Vascular Neurology, Dementia and Ageing Research, Department of Neurology, University Hospital Essen, University of Duisburg-Essen, Hufelandstrasse 55, Germany.

**Keywords:** astrocytes, microglia, brain, development, transcriptomics, neurodegeneration

## Abstract

Glia cells are essential for brain functioning during development, aging and disease. However, the role of astroglia plays during brain development is quite different from the role played in the adult lesioned brain. Therefore, a deeper understanding of pathomechanisms underlying astroglia activity in the aging brain and cerebrovascular diseases is essential to guide the development of new therapeutic strategies. To this end, this review provides a comparison between the transcriptomic activity of astroglia cells during development, aging and neurodegenerative diseases, including cerebral ischemia. During fetal brain development, astrocytes and microglia often affect the same developmental processes such as neuro-/gliogenesis, angiogenesis, axonal outgrowth, synaptogenesis, and synaptic pruning. In the adult brain astrocytes are a critical player in the synapse remodeling by mediating synapse elimination while microglia activity has been associated with changes in synaptic plasticity and remove cell debris by constantly sensing the environment. However, in the lesioned brain astrocytes proliferate and play essential functions with regard to energy supply to the neurons, neurotransmission and buildup of a protective scar isolating the lesion site from the surroundings. Inflammation, neurodegeneration, or loss of brain homeostasis induce changes in microglia gene expression, morphology, and function, generally referred to as “primed” microglia. These changes in gene expression are characterized by an enrichment of phagosome, lysosome, and antigen presentation signaling pathways and is associated with an up-regulation of genes encoding cell surface receptors. In addition, primed microglia are characterized by upregulation of a network of genes in response to interferon gamma. *Conclusion*. A comparison of astroglia cells transcriptomic activity during brain development, aging and neurodegenerative disorders might provide us with new therapeutic strategies with which to protect the aging brain and improve clinical outcome.

Currently, neuroprotective therapies for the aging brain and cerebrovascular diseases are hardly available. Glia cells are essential for brain functioning during development and in the adult brain. Although both cells are fundamentally different in origin and function, they often affect the same developmental processes such as neuro-/gliogenesis, angiogenesis, axonal outgrowth, synaptogenesis, and synaptic pruning. A better understanding of the origin, differentiation process and developmental functions of microglia and astrocytes will help to fully appreciate their role both in the developing as well as in the adult brain, in health and disease. In the adult brain astrocytes are a critical player in the synapse remodeling by mediating synapse elimination. However, in the lesioned brain astrocytes proliferate and play essential functions with regard to energy supply to the neurons, neurotransmission and buildup of a protective scar isolating the lesion site from the surroundings. Likewise, during the prenatal phase, microglia eliminate immature synapses a process called synaptic pruning. In the adult, unlesioned brain, microglia are associated with changes in synaptic plasticity, showing a decrease in the motility of the microglial processes during low neuronal activity. However, inflammation, neurodegeneration or loss of brain homeostasis induce changes in gene expression and microglial morphology and function, generally referred to as “primed” microglia. This change in gene expression is characterized by an enrichment of phagosome, lysosome, and antigen presentation signaling pathways. Therefore, a better understanding of the origin, differentiation process and developmental functions of microglia and astrocytes will help to fully appreciate their role both in the developing as well as in health and disease of the adult brain.

**Table 1:** Neuroglia transcriptomic activity during brain development, aging and neurodegenerative disorders.

Gene symbol	GO	BIOLOGICAL PROCESS	Reference	
ASTROCYTES & BRAIN DEVELOPMENT. RESPONSES TO STROKE			
Mertk	GO:0004714	development; astrocyte-mediated synaptic remodellig; phagocytic pathway	[7][175-177]
Prelp	GO:0005615	prolargin; inflammation; microglia; MMP-9; ECM clearancecell proliferation;	[9][10][11]
Gfap	GO:0014002	fetal brain development; scar buildp post stroke adult brain	[1][15-18]
Gpd1	GO:0006072	glycerol-3-phosphate metabolism; astrocytes; gluconeogeesis; increased after stroke	[20-23]
Slc14a1	GO:0071918	urea transmembrane transport; astrocytes	[24-28]
Mt2A	GO:0046872	astrocytes; inflammation;	[29][30][31][34][35-37]
Nqo2	GO:0003955	astrocyte autophagy; development	[42][43]
MICROGLIA IN BRAIN DEVELOPMENT, HOMEOSTASIS & DEGENERATION			
Il6r	GO:0042531	brain development; microglia, stroke; conversion to a pro-inflammatory phenotype	[65-71]
Cd38	GO:0030890	astrocytes maturation, brain development; perivascular macrophages	[72-77]
RT1-Da	GO:0002469	microglia; IFNγ MHC class II; brain development; brain degeneration	[125]
RT1-DMb	GO:0031902	microglia; IFNγ MHC class II; brain development; brain degeneration	[81][125]
RT1-DMa	GO:0023026	microglia; IFNγ MHC class II; brain development; brain degeneration	[81][125]
Cd4	GO:2000562	CD4+ cells; maturation of fetal microglia; mediators of tissue damage after stroke	[86-88]
BRAIN PARENCHYMA RESIDENT MICROGLIA			
Slc2a5	GO:0015755	monocytes; fructose metabolism; brain development	[95-98]
P2ry12	GO:0045028	microglia; brain homeostasis; LPS/inflammation-activated; increased in the aging brain	[91-94][99-101]
P2ry13	GO:0007186	microglia ADP receptor; brain development and homeostasis	[88][92-94][103]
P2rx4	GO:0004931	microglia ATP receptor; brain development	[104]
Upp1	GO:0044206	Uridine metabolism; monocytes proliferation	[106-109]
C1qa	GO:0006958	complement activation, classical pathway	[89][110-113]
C1qb	GO:0030449	regulation of complement activation	[89][110-113]
C3	GO:0006956	microglia, complement activation after ischemic stroke	[79][89][110-112][114][117][170]
Lyz2	GO:0050829	monocyte-derived microglia; bone marrow monocytes colonizing brain	[114][115]	
Cfh	GO:0030449	regulation of complement activation;	[116-118]
Cx3cr1	GO:0019957	brain development and homeostasis; recruitment to sites of neuroinflammation	[79][88][92-94][110][119-121]
Nfe2l2	GO:0006357	negative regulator of M1 polarization and pro-inflammatory response	[120-123]
PRIMED BRAIN MICROGLIA IN RESPONSE TO STROKE			
CD73/Nt5e	GO:0006196	microglia conversion into a pro-inflammatory phenotype	[130][131]
Ptgs2	GO:0004666	COX-2; inflammation; microglia; microblood vessel	[132-134][137][138]
Mmp9	GO:0030198	ECM degradation brain development; expressed by neutrophils in ischemic stroke	[9-14]
Alox5ap	GO:0004464	activated microglia; leukotriene synthesis by damaged neurons	[175]
Pla2g4a	GO:0047498	microglia; LPA-mediated neuroinflmmation; activation of STAT1 and STAT 3 pathways	[141-144]
Stat1	GO:0060337	microglia to macrophage conversion in response to inflammation	[67][144-146][148-150]
Stat6	GO:0007259	microglia to macrophage conversion in response to inflammation	10.1172/jci.insight.131355 [151]
Ptafr	GO:0007186	microglia; Salivary Evs; inflammation; brain injury	[93][153][154]
Cd53	GO:0005887	Microglia, activated	[76][93][157][158][100][156]
Myo1e	GO:0030050	microglia, phagocytosis, inflammation; vesicle transport	[159-161]
Cd74	GO:0002503	microglia conversion to an inflammatory phenotype; increases aging brain	[25][76][156][162][163]
Il7r	GO:0046427	Il7r mRNA upregulated upon transition from monocyte to macrophage	[164][165]
Irf5	GO:0002376	microglia proinflammatory response; interferon response regulator	[166-168]
Dapp1	GO:0005547	microglia activation; Interferon signaling; downstream	[169][170]
BRAIN MACROPHAGES. RESPONSE TO STROKE			
Itgam	GO:0007229	Cd11b; upregulated peripheral blood ischemic stroke patients	[165][173]
Anxa3	GO:0005544	phagocytic macrophages; calcium-dependent phospholipid binding	[174][175]
MerTK	GO:0004714	microglia, phagocytosis of viable neurons; brain ischemia	[7][176-178]
Slc6a20	GO:0005298	astrocytes and microglia; synaptic plasticity; post-stroke phagocytosis	[179][183]
Litaf	GO:0098560	Macrophages; LPS-induced tumor necrosis factor; endolysosomal pathway	[119][184]
Csf1r	GO:0005011	macrophage colony-stimulating factor receptor activity; brain degeneration	[53][92][185][186][187]
Fcgr2b	GO:0050776	transmembrane signaling receptor; regulation of immune response	[191][192]
Mpeg1	GO:0042742	perforin-2; macrophages; enriched in synapse-rich regions	[191][192]
Chi3l1	GO:0005576	chitinase 3-like protein 1, macrophages-secreted into extracellular region	[15][193]
Arhgap25	GO:0006911	granulocyes; phagosome formation, engulfment	[194-197]
Gpr34	GO:0045028	G protein-coupled purinergic receptor activity; phagocytosis; microglia homeostasis	[92][94][193][198][199]

## 1. ASTROCYTES IN BRAIN DEVELOPMENT AND EARLY TRANSCRIPTOMIC RESPONSES TO CEREBRAL ISCHEMIA IN THE ADULT BRAIN

### 1.1. Astrocyte activity during brain development and early post-natal stages

Astrocytes are the most abundant subtype of glial cells in the central nervous system (CNS). During fetal mouse brain development, the neurogenic-to-gliogenic transition occurs between E12 and E16 while tissue-resident macrophages and microglia develop from the extraembryonic yolk sac and invade the brain before E9.5 [1]

The early astrocytes (approximately, E15) express Gfap, Agt and Aqp4 mRNAs [1]. During the first postnatal week of the mouse cortex, astrocytic activity is coincident with synaptic plasticity and synaptogenesis including formation, maturation, and elimination of synapses by a wide range of newly identified secreted and contact-mediated signals [2, 3]. Indeed, addition of astrocytes to neuronal cultures was sufficient to promote synapse formation and spontaneous activity of RGC neurons, which are largely inactive in the absence of glial support [4-6].

More recent studies have revealed a novel role for astrocytes in mediating synapse elimination and identified *Mertk* gene as a critical player in synapse remodeling in the developing and adult mouse brain [7](Table 1). Similarly, developmental astrocytes express *Prelp* mRNA, encoding prolargin (PRELP), a leucine-rich repeat protein that is present in connective tissue. Its major function is to anchor type I collagen to the basement membranes. PRELP is expressed in astrocytes, pericytes, vascular smooth muscle cells and tumor-associated macrophages [8] and is a substrate for metalloproteinase 9 (MMP9) which is most likely involved in extracellular matrix remodeling during brain development and psoriatic arthritis [9-11]. Indeed, a recent study has shown that neutrophils rely on MMP9 and MMP13 for a rapid and orderly migration response to brain injury in mice [12, 13](Table 1).

**Figure 1.**
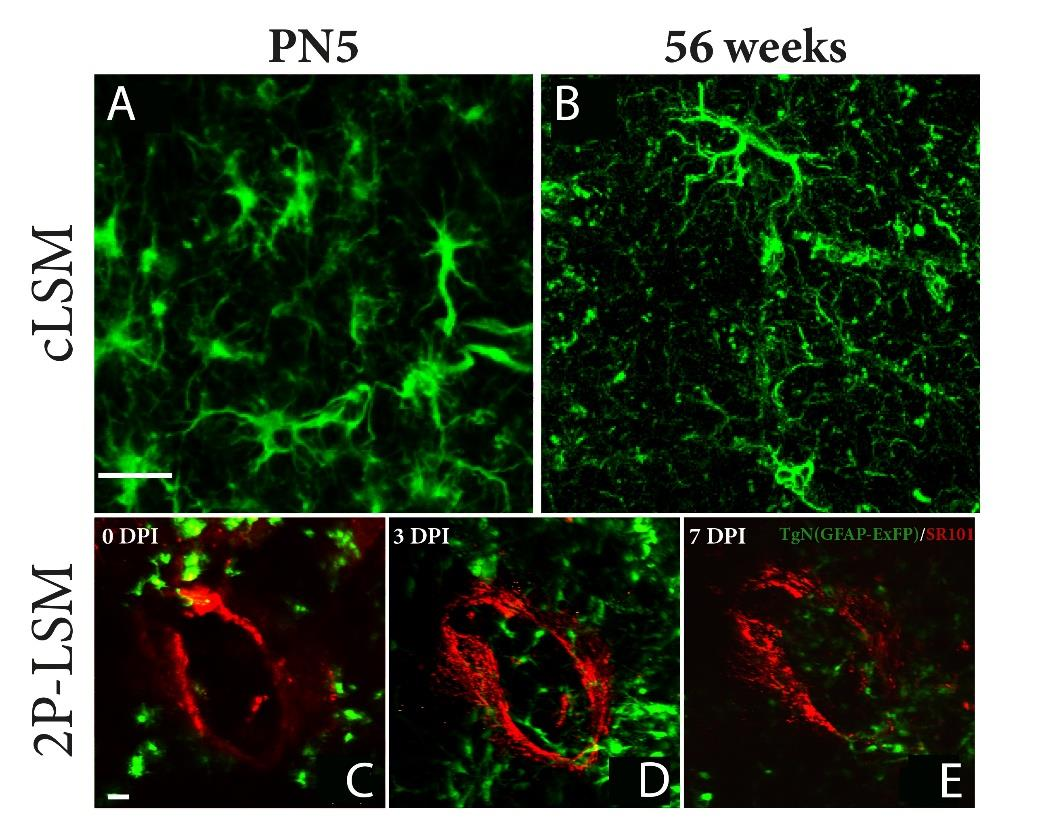
Laser scanning microscopy of cortical mouse astrocytes at different ages. (A) cLSM images of cortical astrocytes obtained at postnatal day5 (5 PN) shows cells with short, densely packed branches, through the cortex. At this time point astrocytes seem to be less ramified, with shorter branches compared to (B) adult astrocytes that display more and longer branches covering a larger volume of the microenvironment. (C) *In vivo*, 2P-LSM, shows astrocytes that react slower to changes in their microenvironment, even if that change is a large one (red: 500 µm stab wound injury). (D) It takes astrocytes up to 3 days post injury (DPI) to isolate the necrosis area by forming a physical barrier around the lesion. (E) At 7 days astrocytes are forming a packed scar tissue around the lesion. Scale bar 37 µm.

A wealth of evidence show that MMP-9 activity is critical for the CNS development. In particular, MMP-9 plays a role in the development of sensory circuits during early postnatal periods, called “critical” periods [14]. Further, survival predictors based on gene signatures for glioblastoma obtained from microarray experiments achieved 100% accuracy if composed of *Gfap*, *Pzprz1*, *Gpm6b* and *Prelp* transcripts suggesting that PRELP is involved in extracellular matrix (ECM) remodeling in glioblastoma. Similarly, a collection of *Gfap*, *S100b*, *Aqp4* and *Chl3l1* mRNAs [15] has been consistently associated with astrocytes-associated ECM remodeling during prenatal human brain development, aging and plaques at sites of Aβ deposits in the AD brains [15-17] (Table 1).

### 1.2 Transcriptomic activity of astrocytes in the adult brain and following injury

Following cerebral ischemia, astrocytes play a distinct role from that they played during development. The upregulation of the astrocytic marker GFAP in response to cerebral insults is well documented (Fig. 1). Thus, proliferative, GFAP-expressing astrocytes are major contributors to the glial scar confining the infarcted area, especially in aged brains [18]. Quite interesting, astrocytes in the tissue adjacent to the infarct core showed perifocal caspase-3 expression that was localized to the nuclear compartment. However, the astrocytes showed no apoptotic signs such as fragmentation and condensation suggesting a predominantly non-apoptotic role of caspase early after stroke [19].

Neurons require enormous energy to maintain a balanced functional activity. Compared to other cell types, neurons possess peculiar features due to the presence of a metabolic coupling between astrocytes and neurons. Thus, glucose is taken up by astrocytes and converted to dihydroxyacetone phosphate and further to ATP, a process requiring the enzyme glycerol 3-phosphate dehydrogenase encoded by *Gpd1* mRNA. Glycerol-3-phosphate dehydrogenase (GPD1) is an enzyme that catalyzes the conversion of dihydroxyacetone phosphate to glycerol 3-phosphate and further to glycerol and NAD^+^ and serves as a major provider of glucose via gluconeogenesis and reflects an adaptation of energy metabolism to the acute lack of glucose. During brain development, the amount of glucose provided by maternal circulation and taken up by neurons is low [20, 21]. Therefore, there is no need of gluconeogenesis during brain development and as a consequence, the GPD1 levels are low. However, upon the onset of ischemia, in order to meet the higher energy demand, compensatory pathways are initiated and the brain shifts the cellular machinery from aerobic to anaerobic metabolism that leads to vast increases in the levels of GPD1 which is involved in mitochondrial reoxidation of glycolysis-derived NADH in a rat model of stroke [22, 23].

Even more energy can be provided by an increased utilization of aminoacids alanine and glutamine to provide ATP and thus compensate for the limited energy supply caused by cerebral ischemia. However, an increase in aminoacids metabolism will result in an increased nitrogen concentration in neurons that has to be removed as urea. *Slc14a1* is a gene encoding a urea membrane transporter in astrocytes that is co-expressed with the astrocytic marker GFAP and is important for the removal of urea from the CNS [24]. Moreover, enhanced expression of the *Slc14a1* gene has been reported in conditions favouring urea accumulation in neurodegenerative diseases, including Alzheimer's and Huntington's disease [25-27]. An increased expression of *Slc14a1* mRNA in the infarcted cortex of mice subjected to ischemic stroke and traumatic brain injury has been also reported [28] (Table 1).

Astrocytes express a number of essential proteins in the CNS, including metallothioneins (MTs). Glial cells of the human fetal brain express MTs in the gray matter and blood vessels starting with week 35 and their most likely function is to regulate the intracellular concentration of metal ions during brain development [29]. Alternatively, MT is thought to be actively secreted by astroglia and picked up by neurons through the LRP-2 (megalin) and the LRP-1 receptor [30].

CREB is one of the major regulators of neural precursor cells differentiation, neurotrophin synthesis during development and synaptic plasticity [31]. In the adult, unlesioned brain, MT-mediated activation of megalin receptor triggers intracellular activation of transcription factors and the cAMP response element binding protein (CREB) targeting the phosphoinositide 3-kinase pathway. However, insults to the brain induce reactive astrocytes to express high levels of MTs after brain ischemia as well as in the brains of epilepsy and AD patients and may represent a cellular defense response to inflammatory signals [32-35]. Indeed, *Mt2a* mRNA was identified as the most significant induced transcript in the early phase of the ischemic stroke in mice making the metallothionein family of proteins a very promising candidate for a future neuroprotective stroke therapy [33].

Recent human genetic and genomic evidence has demonstrated an emerging, significant role of autophagy in human brain development. Indeed, autophagy genes, such as *ALFY*, *Atg9* and *Atg1* control synaptic plasticity by regulating axon guidance, synaptic outgrowth and formation. In the adult brain autophagy genes ATG5 and AYG7 regulate neurotransmission [36-38].

During development, autophagy-related gene *Atg5* is essential for astrocyte differentiation in the developing mouse cortex [36]. Astrocytes protect neurons from oxidative stress induced by hydrogen peroxide, toxic metabolites, and neurotransmitters, including glutamate, dopamine and 6-hydroxydopamine by several mechanisms including increased autophagy [37]. Indeed, autophagy is the core regulator of CNS plasticity and neurodegeneration [38].

**Figure 2.**
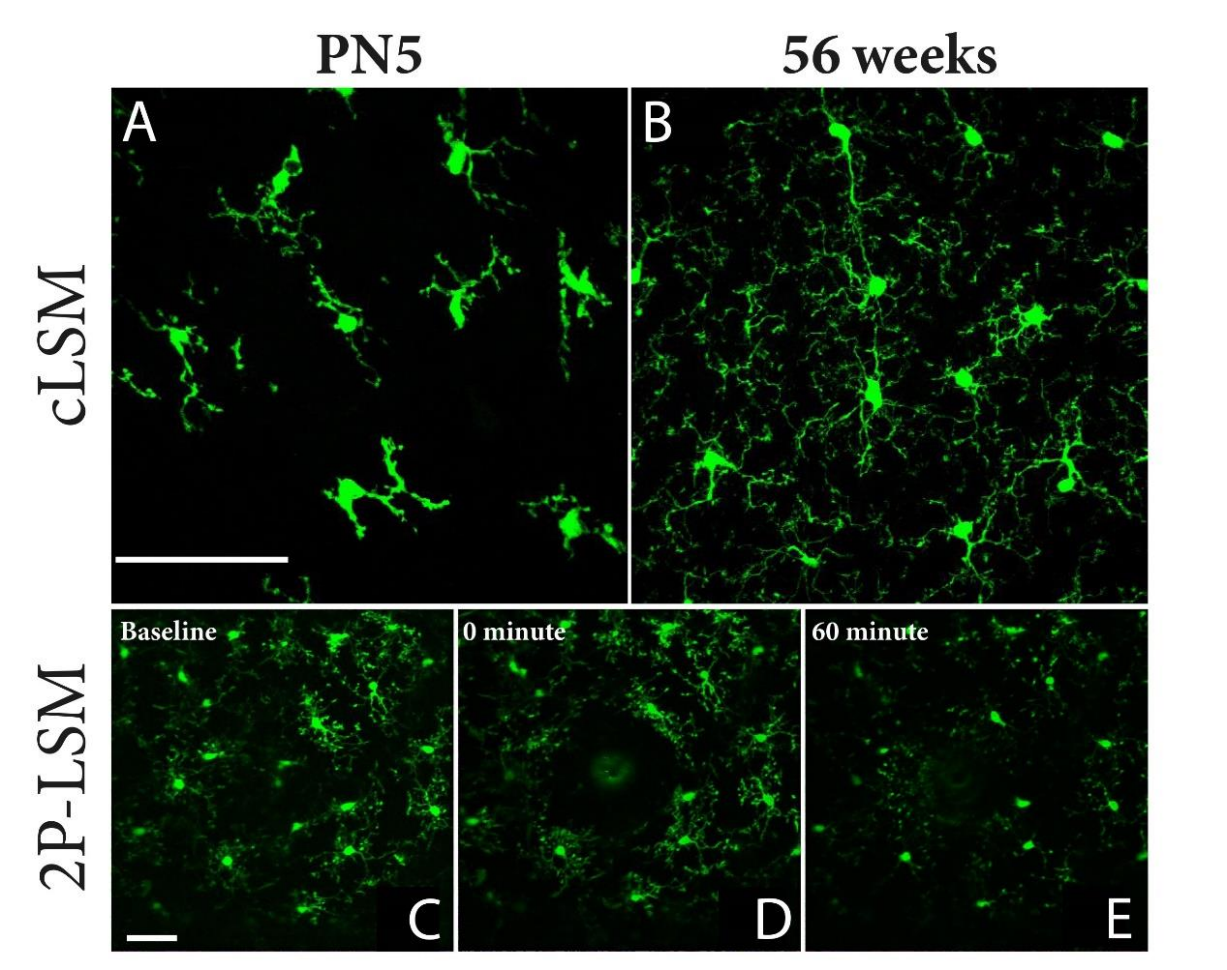
Laser scanning microscopy of cortical mouse microglia at different ages. (A) Confocal laser scanning microscopy (cLSM) images of cortical microglia obtained at 5 days postnatal (5PN) shows a lower number of microglia but equally spread through the cortex. At this time point microglia seem to be less ramified, with thinker branches compared to (B) adult microglia that display more and thinner branches covering a larger volume of the microenvironment. (C) *In vivo*, 2 photon laser scanning microscopy (2P-LSM), shows microglia constantly scanning their microenvironment, and (D) once detected, they start isolating the laser induced micro-lesion lesion by extending their branches towards the lesion and retracting the ones opposed of the lesion in a process called polarisation. (E) For small lesions, within 60 minutes microglia are able to isolate it completely. Scale bar 75 µm.

However, oxidative stress limits the neuroprotective activity of astrocytes, an effect that has been invoked in the progression of neurodegenerative disorders, including Parkinson disease (PD) [39]. The inhibitory effect of autophagy on the oxidative stress is in turn mediated by the quinone reductase (NQO2), and can be prevented by the specific NQO2 inhibitor, NMDPEF, that restores autophagy in treated astroglial cells [40]. Indeed, inhibition of NQO2 is neuroprotective and is also a primary therapeutic target after stroke [41] (Table 1). A cartoon depicting the role of astrocytes in the healthy and injured brain is shown in Fig 2.

## 2. MICROGLIA CELLS IN BRAIN DEVELOPMENT AND THEIR NEW ROLE IN THE POST-ISCHEMIC ADULT BRAIN

Microglia are the only resident immune cells of the CNS and as such are involved in almost all CNS pathologies. At cellular level, their function can range from engulfing apoptotic cells [42] and phagocytosis of accumulating extracellular debris [43] to repairing damaged brain parenchyma [44, 45]. These roles are essential in the normal function of the CNS and show their involvement in maintaining brain microenvironment and CNS homeostasis [46, 47].

Extensive research has been carried out to elucidate the origin of microglia. The original suggestion of Rio Hortega's that microglia are of mesodermal origin has been recently confirmed [48, 49]. Thus, we now know that starting with day 9 of embryonic life, primitive macrophages originate from c-kit+ yolk sac progenitors, in a PU.1- and IRF-8-dependent manner [50] and start to migrate towards the brain [51]. This process is rapid and by the 10-11 days of embryonic life microglia are present in the cephalic mesenchyme and neuroepithelium where they start to proliferate [52] and populate all major compartments of the brain and spinal cord [53].

While the origin of microglia is now firmly established, the microglia dynamics over the lifespan is still largely unknown. This has been made even more difficult by the fact that we do not fully understand how the microglia population is maintained. Some studies suggest that the entire microglial population slowly renews itself several times during its lifespan [54], as a result of intense cell proliferation rather than infiltration of blood macrophages, which further points to the difference between microglia and other tissue-resident macrophages [55]. Hence, we failed to understand whether the different phenotypes displayed by microglia [56] with aging are a result of intrinsic changes or the result of imperfect cellular integration.

In addition to their involvement in pathology, microglia have been implicated in some essential physiological processes. These roles are mainly related to neural plasticity and activity and vary over the lifespan. During the prenatal phase, microglia eliminate immature synapses a process called synaptic pruning [57]. In the adult brain, microglia are associated with changes in synaptic plasticity, showing a decrease in the motility of the microglial processes during low neuronal activity or following deprivation of visual experience, suggesting a bi-directional neuron-microglial relationship [58-60].

### 2.1 MICROGLIA IN BRAIN DEVELOPMENT, HOMEOSTASIS AND DEGENERATION

Brain resident microglia derive from yolk sac primitive macrophages, which enter and colonize the developing brain at about E9.5 [51]. However, as BBB formation is completed by day E13.5, microglial population is maintained by self-proliferation. Even if blood monocytes enter the brain following BBB damage, they do not contribute significantly to the resident CNS microglia [61].

### 2.1.1 Perivascular Microglia/Macrophages

Recently, a population of embryonically derived macrophages with a microglia-like phenotype located at the brain-circulation interface has been described. These non-parenchymal brain macrophages, in contrast to the resident microglia brain parenchyma, include meningeal, perivascular, and choroid plexus macrophages and maintain their population by self-renewal [49, 62].

Pro-inflammatory cytokines TNF-α, IFNγ, IL-1β, and IL-6 are major inducers of immune activation after brain insults, both in the peripheral and central immune systems. Thus, following oxygen deprivation, perivascular microglia release IL-6 and initiate IL6r-mediated signaling converging on JAK/STAT3 pathway at the lesion site [63-65](Table 1). However, in the developing and adult brain, IL-6 and its receptor IL-6R, promote proliferation of neuronal precursor cells, NSCs. Thus, IL-6 signaling is both necessary and sufficient for adult NSC self-renewal and has long-lasting effects on the size of adult NSC pool [66].

A number of studies have shown an increase in *Il6r* mRNA expression early after cerebral ischemia both in neurons and microglia [67]. Indeed, IL-6R expression has been reported to be restricted to neurons of the post-ischemic brain [68]. Furthermore, a recent study showed that activated microglia will induce Il6R expression in neurons and may have even beneficial effects on neuronal survival [68, 69].

CD38 is a multifunctional transmembrane protein that possesses ADP-ribosyl cyclase activity. This enzyme produces cyclic ADP-ribose from nicotinamide adenine dinucleotide (NAD) and releases Ca^2+^ from intracellular stores. During development, CD38 is required for astrocyte differentiation [70] while in the adult brain it has been associated with oxytocin release and social behaviour [71]. An increase in CD38/MHCII+ cells has been also recently reported in perivascular microglia of the aging mouse brain [72].

Neuroinflammation significantly increased CD38 expression especially in the rodent hippocampus and inhibition of CD38 or supplementation of nicotinamide riboside ameliorated lipopolysaccharide-induced microglial and astrocytic neuroinflammation [73]. Furthermore, an increase in *Cd38* mRNA in perivascular macrophages has been also noted in response to ischemic stroke in stroke patients and in a mouse model of stroke [74]. Of note, an increased expression of the *Cd38* transcript in NAD+ deficient mice suggests that after stroke *Cd38* mRNA could act in concert with the *Gpd1* transcripts from astrocytes to increase glucose availability [75].

The surprisingly involvement of the major histocompatibility complex class I (MHC-I) molecules in regulation of neurite outgrowth, establishment and function of cortical connections and long-term homeostatic plasticity are now well established in the brain [76, 77]. Importantly, MHC-I molecules may also be involved in maintaining brain connectivity and synaptic function by modulation of neuronal and spine morphology during non-pathological aging [78].

However, the role of MHC-II molecules in brain wiring and synaptic plasticity during brain development is less well known. More recent results suggest, nevertheless, that the expression of the major histocompatibility antigens class-2 (MHC-II; RT1-D1, RT1-DMa, and RT1-DMb) act as cell adhesion molecules that are expressed early in human neural stem cells both *in vitro* and *in vivo* [79].

In the postnatal brain, these molecules are downregulated. However, following ischemic stroke, MHC-II molecules are re-expressed by microglia/macrophages [80]. A similar upregulation has been reported in the activated microglia of the aging brain [81] and experimental allergic encephalomyelitis [82, 83] (Table 1).

Similar to the lymphatic drainage system of the body, the CNS is drained by the cerebrospinal fluid (CSF), that is generated in the choroid plexus from arterial blood and returned to the venous blood at the granulation villi of the arachnoid membrane. The CSF contains CD4+ and, to a lesser extent, CD8+ T cells, which patrol the borders of the CNS and provides immune protection. Using a combination of imaging, single cell, and surgical approaches, a CD69+/CD4-T cell population distinct from circulating CD4-T cells, has been recently identified. Furthermore, resident CD4-T cells were required for proper maturation of fetal brain resident microglia [84]. Indeed, using post-mortem brain tissue, it was recently shown that the human brain harbors both CD8+ and CD4+ cells.

According to current understanding, early ischemic injury is mediated primarily by infiltrated lymphocytes and innate-like lymphocytes [85]. Indeed, CD+ cells depletion after experimental stroke inhibited B cell infiltration into the brain and improved cognitive functions [86] (Table 1).

### 2.1.2. BRAIN PARENCHYMA RESIDENT MICROGLIA INVOLVEMENT IN ISCHEMIC STROKE

In the unlesioned adult brain, microglia fulfill homeostatic functions including sensing the brain microenvironment, removing cellular debris by phagocytic scavenging, remodeling the brain circuits through synaptic pruning and neuronal plasticity and maintaining oligodendrocyte progenitors thus contributing to myelinogenesis. Indeed, microglia depletion in the adult brain leads to disturbance in memories due to lack of the complement-dependent elimination of synapses [87]. Furthermore, resident microglia assist sprouting of capillaries by contacting the vasculature in areas not covered by astrocytes, ultimately enhancing memory and learning [88]. Thus, depletion of microglia postnatally reduces the glutamatergic excitatory synapse function and leads to learning task deficits [89]. In the early post-natal mouse brain, the genetic signature of fetal microglia changes during the transition to resident microglia, a process that is assisted by brain resident CD4 T cells [84]. Thus, single-cell sequencing revealed that in the absence of murine CD4 T cells, resident microglia remained suspended between the fetal and adult states. Further, although developmental functions of microglia from MHC II KO mice were seemingly intact, a maturation defect resulted in alteration of key functions of microglia, including failure of synaptic sprouting and behavioral abnormalities [84]. Quite interesting, injury to the adult brain reversed the genetic signature of microglia to that of the prenatal genetic signature [84].

RNA-seq analysis identified microglia-specific markers involved in sensing and housekeeping pathways which include *Apbb1ip*, *Cx3cr1*, *C1qa*, *C1qb*, *C3*, *Cfh*, *Csfr1*, G*pr34, Lyz1, Lyz2, Mpeg1, Nfe2l2, P2rx4, P2ry12, P2ry13, Ptafr, S100A8, S100A9, Slc2a5, Trem2, Tmem119* and *Upp1* transcripts [90-92] (Table 1).

Microglia also play a role in the regulation of metabolism by sensing the levels of brain metabolites via the *Slc2a5* and *P2ry12* transcripts that are highly expressed in brain-colonizing monocytes during brain development and to be then downregulated in the adult brain [93]. *Slc2a5* mRNA encodes for solute carrier family 2 member 5 (SLC2A5), a fructose/glucose transporter primarily expressed in microglia within the CNS [94]. Therefore, a vast increase in the number of proliferating microglia after stroke leads to increased intracellular levels of glucose. Indeed, the majority of acute stroke patients have disorders of glucose metabolism which, along with other comorbidities, will cause delays in the recovery of brain function after stroke [95, 96].

*P2ry12* mRNA encodes for the purinergic receptor P2RY12 that is expressed in non-activated microglia in the developing mouse brain, being involved in the directed motility of microglial processes to sites of damage where ATP/ADP is released [97]. P2RY12 has an increased expression in the microglia of the aged mouse brain as well as in the brains of AD patients [98, 99]. A prominent expression has also been reported following lipopolysaccharide (LPS)-induced brain inflammation in animal model [99]. Further, immunofluorescence imaging of P2Y12-positive microglia in the cortex of sham and mice subjected to traumatic brain injury (TBI) has shown that P2Y12-positive microglia was converted into activated microglia following the release of the inflammatory cytokines IL-1β, TNF-α, CCL2, IL-6, at 24 h post-injury [100].

Transcriptome data suggest that P2RY13 is the second most expressed neurotransmitter receptor in resting microglia. The *P2ry13* transcript encodes the ADP-activated P2Y13 receptor which under basal conditions is part of a signaling pathway whereby microglia contribute to the homeostatic control of adult hippocampal neurogenesis via a nucleotide-mediated mechanism. However, in the adult brain *P2ry13* mRNA is absent from neurons, astrocytes, and neural progenitor cells. Intriguingly, disruption of *P2ry13* gene expression in P2ry13 KO mice, increased the number of progenitor cells and newly formed neuron [101]. Likewise, *P2ry13* transcripts were found to be upregulated in the ischemic hemisphere as compared to the unlesioned, contralateral hemisphere of mice [86].

The *P2rx4* transcript is expressed at low levels in neurons and glial cells of the adult CNS. P2X4 is activated by ATP and contributes to synaptic transmission and synaptic plasticity. Of note, the activation of P2X4R by tissue injury activates microglia opening the calcium influx channels on the cell membrane that leads to activation of AKT and JNK signal pathways culminating in the release of pro-inflammatory cytokines TNF-a, IL-18, IL-10 and finally causing neuronal damage [102].

Pharmacological inhibition of P2X4R in an animal model of stroke was shown to be protective by reducing the number of infiltrating pro-inflammatory myeloid cells and improved both acute and chronic stroke recovery by reducing the levels of interleukin-1 beta (IL-1β) and limited the blood brain barrier (BBB) permeability to the leakage of leukocytes into the infarct territory at 3-day post-stroke [103].

*Upp1* mRNA encodes an enzyme required for uridine metabolism. It is specific for proliferating monocytes during brain development [104]. In brain glioma, UPP1 was associated with immune and inflammatory response and higher UPP1 levels correlated significantly with a shorter survival time. More specifically, UPP1 was particularly associated with MHC-II and LCK, which were mainly associated with activities of antigen-presenting cells and T cells [105]. UPP1 has also been found in the perihematomal tissue after intracerebral hemorrhage [106] and in a mouse model of transient middle cerebral artery occlusion [107].

Immune system components also regulate synapse formation and refinement during neurodevelopment. Resident microglia play an active role in synaptic surveillance and remodeling in the developing and adult brain through the neuronal expression of “Eat Me” signals C1q, C3 and CX3CL1 as well as expression of the complement receptor CR3/CX3CR1 signaling in microglia [108].

In humans, complement *C1qb* and *C3* transcripts rise early during neurodevelopment and are highest in toddlers to decline then in teenagers [109]. Likewise, the expression of the microglial complement receptor subunit *Cd11b* mRNA, is increased early in life and peaked early in brain development (1-2 years) [109].

Complement components have been involved not only in shaping brain wiring during development but also in brain degeneration in Alzheimer’s and Huntington’s diseases [110]. The robust increase in the expression of C1Q rat brain microglia and cerebrospinal fluid after an ischemic insult is well documented [111]. Likewise, *C3* and *Lyz2* transcripts upregulation after focal ischemia is a key constituent in complement-related inflammatory tissue injury most likely via a C3a anaphylatoxin-mediated mechanism [112, 113].

C3 conversion to C3a is controlled by the Complement factor H encoded by *Cfh* mRNA. CFH is a soluble complement regulator that is essential for controlling the alternative pathway in the blood. However, in the brain, several miRNA species, including miR-125b and miR-146a, have been found to target the mRNA coding for complement factor-H resulting in a decrease in the CFH expression in the brains of AD patients [114]. In the inflamed, ischemic brain, CFH is involved in the complement-mediated clearance of apoptotic and damaged cells [115, 116].

Proteins of the innate immune system, C3 and its receptor CX3CR1 on microglia, play a non-immunological role and are essential for the establishment, function, and wiring of the nervous system mediated by the phagocytic activity of microglia via CX3CR1 that is present on the surface of microglia cells both during development and on activated microglia and macrophages [77].

In the adult, unlesioned brain, CX3CR1, TREM2, progranulin and the scavenger receptor pathways promote clearance of injurious stimuli and act as housekeeping pathways to keep the microglial inflammatory response under control. In a mouse model of ischemic stroke, C*x3cr1* transcripts were found to be upregulated in the ischemic hemisphere as compared to the unlesioned, contralateral hemisphere [86]. Of note, *Cx3Cr1* genetic deficiency prevented the proliferation of CNS microglia and the recruitment of monocyte-derived macrophages from the circulation. Furthermore, in the brains of the *Cx3Cr1*-/- mice neuronal death is mediated by CX3CR1 receptors and the mice displayed significantly smaller infarcts and less severe neurological deficits as compared to wild type controls [117, 118].

Finally, the CX3CL1-CX3CR1 pathway activates the expression of *Nfe2l2* mRNA that encodes the nuclear factor, erythroid 2 like 2 (NFE2L2, NRF2), a transcription factor that regulates several antioxidant enzymes and plays a central role in the inflammatory response [119]. NRF2 is evolutionary conserved and is expressed early in brain development. In the adult brain, however, it has been involved in various human disorders, including upregulation in response to brain ischemia and gliosis in the adult brain [120]. Indeed, NFE2L1(L) functions as a negative regulator of M1 polarization and pro-inflammatory response in microglia. Consequently, its silencing primes microglia towards M1 polarization [118]. The transcription NFE2L2 factor also acts as a regulator of gene expression in autophagy and its deficiency leads to impaired amyloid β precursor protein (ßAPP) processing in a mouse model of AD [121].

### 2.1.3 PRIMED BRAIN MICROGLIA IN RESPONSE TO INJURY

**Figure 3.**
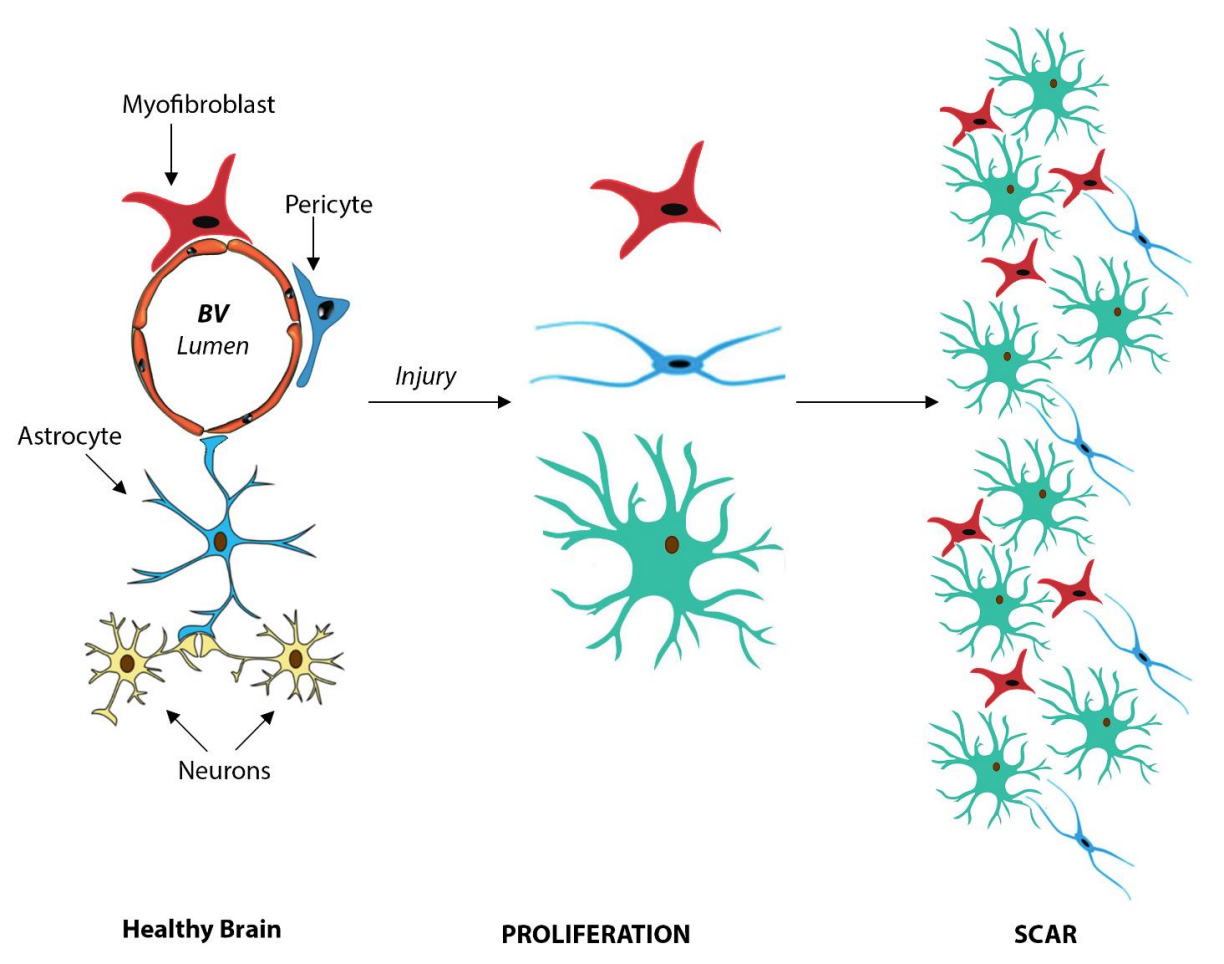
Cartoon depicting the role of astrocytes in the healthy and injured brain. Please note cellular proliferation after injury and buildup of the fibrotic scar tissue by activated astrocytes, myofibroblasts and pericytes.

Resident microglia play an active role in synaptic remodeling in the adult brain [108, 122] However, insults to the brain, inflammation, neurodegeneration or decline in brain homeostasis induce changes in gene expression and microglial morphology and function, generally referred to as “primed” microglia [123](Fig. 3). This change in gene expression is characterized by an enrichment of phagosome, lysosome, and antigen presentation signaling pathways and is associated with an up-regulation of genes such as *Cd53*, *Ptgs2*, *Alox5ap*, *Naaa* and *Il7r* encoding cell surface receptors (Table 1).

Physical exercise and manipulation of glycolytic enzymes by drugs aimed at shifting the metabolic profile of microglia from glycolysis towards oxidative metabolism promote an anti-inflammatory phenotype and enhanced phagocytosis [124-127].

Accumulating evidence demonstrates that metabolic reprogramming acts as a key driver of microglial immune response. Following acute stroke, there is a rapid depletion of ATP and a change in the use of glucose by oxidative phosphorylation to the much less efficient glycolytic-based energy metabolism in the area supplied by the middle cerebral artery. As a consequence, ecto-5′-nucleotidase (CD73)-mediated adenosine formation stimulates microglia conversion to a pro-inflammatory phenotype [128]. Indeed, in a mouse model of glioma, *Cd73* genetic depletion prevents the conversion of microglia to a pro-inflammatory phenotype [129].

Neurodevelopmental and neurodegenerative diseases have been associated with disturbances in brain lipid composition and related enzyme activities [130, 131]. Thus, the polyunsaturated arachidonic and docosahexaenoic acids (AA and DHA) participate in cell membrane synthesis during neurodevelopment, neuroplasticity, and neurotransmission. Indeed, genes involved in arachidonic acid metabolism like cytosolic phospholipases A2 (cPLA2) and cyclooxygenases (PTGS1 and PTGS2) are highly expressed during brain development [132] while the healthy aging brain does not show upregulation of *Pla2g4* or *Ptgs2* mRNAs expression. However, PLA2G4 and PTGS2 are elevated in the post-mortem brains of AD patients [132]. Of note, specific knockdown of *Ptgs2* expression in a mouse model of stroke provided neuroprotection in ischemic stroke by inhibiting apoptosis and promoting proliferation, migration and angiogenesis of endothelial precursor cells [133]. Likewise, pharmacological inhibition of COX-2 limits BBB damage by reducing MMP-9 activity in a mouse model of ischemic stroke [134]. Of note, BBB leakage as late as 1 year after the global ischemic episode would allow the infiltration of CD4^+^ cells into brain parenchyma on a background of persistent activated microglia[135]. Likewise, even after 2 years of global ischemia in animal model, there was significant activation of astrocytes throughout the brain, while microglial activation was found only in the CA1 and CA3 areas as well as the motor cortex [136]. Thus, restoration of BBB integrity could be the first step to limit the damage inflicted by brain hypoperfusion.

Leukotrienes (LTs) are released primarily by neurons expressing the enzyme 5-lipoxigenase (5-LOX) and contribute to the neuropathology of chronic neuro-degenerative disorders by mediating neuroinflammation and neuronal death via the microglial expression of arachidonate 5-lipoxygenase activating protein (ALOX5AP) which anchors 5-Lipoxygenase (5-LOX) to the membrane and thus mediates the contact to the substrate arachidonic acid, culminating in LTs synthesis [137]. Of note, disruption of the *Alox5ap* gene ameliorates focal ischemic stroke by impairing leukotriene biosynthesis [138].

The *Pla2g4a* transcript is expressed following full activation of microglia in response to brain injury to stimulate phagocytic activity and lipid metabolism pathways that generate components of the lysophosphatidic acid (LPA) family. Components of the LPA family are lipid mediators that are indispensable for brain development and function of the nervous system. Thus, LPA is found in the embryonic brain, choroid plexus, meninges, and neural tube and modulates microglia proliferation and membrane ruffling, a pre-requisite of the phagocytic activity [139-141]. For example, lipopolysaccharide-induced systemic inflammation caused a significant increase in LPA concentration and cytosolic phospholipase A2 (*Pla2g4a*) gene expression as well as phosphorylation of the proinflammatory transcription factors, STAT1 and STAT3 in FACS-sorted microglia [142].

The JAK/STAT pathway plays an essential role in cytokine receptor signaling. In the developing brain, JAK-STAT signaling has been involved in neuronal and glial differentiation [143]. More recently, it has been suggested that STAT3 is necessary and sufficient for astrocyte differentiation whereas STAT1 is dispensable [144]. Furthermore, STAT1 and STAT3 have been associated with M1 microglia phenotype resulting in the release of pro-inflammatory cytokines and promoting secondary brain damage in a mouse model of ischemic stroke [145-148].

Another transcription factor of the STAT family is *Stat6*. In the brain, STAT6 phosphorylation is induced by interleukin-4 (IL-4), a key factor that mediates the neuro-immune crosstalk between injured neurons, immune cells, and stem cells. IL-4 also mediates neuronal survival and microglial dynamics during neurodegeneration [149]. Indeed, the STAT6/Arg1 pathway modulates microglia/macrophage phenotype while activation of STAT6 has been reported in macrophages around the ischemic lesion early after experimental stroke in mice but also in stroke patients. Further, deficiency of STAT6 in animal model resulted in reduced clearance of degenerated neurons and enlarged infarct volume [150].

Platelet-activating factor receptor (PTAFR/PAFR) is a pleiotropic phospholipid encoded by the *Ptafr* transcript that is expressed by endothelial, microglia and neuronal cells with proinflammatory, procoagulant and angiogenic properties mediated mainly by the vascular endothelial growth factor expressed on the vasculature. It was also detected in the peripheral blood, cerebrospinal fluid, entorhinal cortex, hippocampus, and temporal cortex of AD brains suggesting that PTAFR could be used as a potential biomarker of CNS inflammation [151]. Of note, an improved neurological deficit and neuroprotection has been reported in mice deficient in PAFR synthesis. Finally, miR-98 can inhibit the microglial phagocytosis of “stress but viable” neurons after ischemic stroke through downregulating PTAFR [152].

Resident microglia play an active role in sensing the environment in the young-adult brain [90-92, 153]. Quite interesting, 81% of the genes involved in sensing are downregulated during aging of the brain. Indeed, a transcriptomic profile of the aged human brain provided a signature of 1148 genes detected in cortex, including *Cd53* mRNA that encodes a brain microglia cell surface antigen, a protein with four membrane-spanning domains that belongs to the tetraspanin family that mediates signal transduction in macrophages.

A recent study done on a large number of human brain tissue obtained at autopsy has revealed that CD53 immunoreactivity increases, along with microglia activity, in the aging brain [98, 154]. Quite recently, a highly significant increased expression of CD53 has been reported in atherosclerotic plaques [155] and neurofibromatosis type 2 vestibular schwannomas [156]. Of note, *Cd53* mRNA also showed high levels of expression up to 21 days after cerebral ischemia in mice [74].

Likewise, the *Myo1e* gene that encodes for a nonmuscle MYOSIN IE, a structural protein expressed in activated astrocytes, has been identified in the glial cells surrounding the Aß plaques in the brains of AD patients [157] as well as in a rat model of cerebral ischemia [158]. More recently, it has been shown that MYOSIN 1E promotes the expression of genes that are critical for the proinflammatory response in microglia [159]. Of note, MYOSIN1f, that is closely related to MYOSIN 1e, is required for neutrophil migration in the infarcted area of the *Myosin1f* ^-/-^ mice [38].

In the adult brain, *Cd74* mRNA codes for a membrane receptor for the cytokine macrophage inhibitory factor (MIF) which promotes conversion of microglia to the M1 pro-inflammatory phenotype via MIF released by injured neurons in the ischemic brain [160], [25],[161]. A recent study done on a large number of brain tissue prelevated at autopsy from aged humans revealed that CD74 expression increased along with microglia activity [154]. Of note, an increase in *Cd74* transcripts has been also noted in response to neuroinflammation in a mouse model of stroke [74].

Primed microglia are characterized by upregulation of a network of genes in response to interferon gamma, including *Il7r, Irf5*, *Dapp1*, *Ifi27*, *Sp100* and MHC II genes coding for RT1-Da, RT1-DMa, RT1-DMb [123, 153]. *Il7r* mRNA encodes the interleukin 7 receptor (IL7R). An i*n vivo* study focused on tracing the lineage of cells with an expression history of *Il7r* mRNA, reported that within the fetal tissue, IL7R regulates tissue-resident macrophage during fetal development by upregulation of *Il7r* mRNA expression during the transition from monocytes to macrophages [162]. However, in adults, IL7R has been reported to decrease in the peripheral blood of ischemic stroke patients [163].

*Irf5* mRNA encodes the interferon regulatory factors (IRFs) that mediate macrophage activation in peripheral immune cells [164]. Thus, microglial expression of interferon regulatory factor 5 (IRF5) has been linked to proinflammatory responses to cerebral ischemia [165]. Using genetic inactivation of both *Irf4* and *Irf5* genes, they have shown that IRF5 signaling directs the microglial proinflammatory response while IRF4 signaling activates the microglial anti-inflammatory action [165].

Aging has a significant effect on the immune response in mice subjected to cerebral ischemia. Thus, a comparison between young (9-12 weeks) and aged (18 months) male mice subjected to focal ischemia has revealed that young mice had significantly more IRF4/CD206 double positive microglia than aged ones whereas the aged mice had more IRF5+ and MHCII+ pro-inflammatory microglia than young mice [166].

*Dapp1* mRNA (also called *Bam32*) encodes for Dual Adapter for Phosphotyrosine and 3-Phosphotyrosine And 3-Phosphoinositide. The *Dapp1* transcript is downregulated during brain development [167] and re-expressed by activated microglia as part of interferon signaling pathway during brain degeneration [167] as well as following transient focal cerebral ischemia in 3 mo- and 12 mo-old male spontaneously hypertensive rats, along with complement components (C3, C4a) and interferon response regulator (IRF7) [168].

### 2.1.4 BRAIN MACROPHAGES RESPONSES TO STROKE

Early after stroke onset, microglia proliferate and become activated. Microglia activation is mainly characterized by migration to the injured area to switch to a phagocytic phenotype.

During the acute phase of ischemic stroke, the *Itgam/Cd11b* transcripts are highly expressed in activated macrophages/microglia and infiltrating leukocytes of the inflamed brain. Indeed, CD11b is heavily expressed on polymorphonuclear neutrophils (PMNs) that accumulate within capillaries and venules of the ischemic brain territory within the first hours after ischemic stroke followed by their extravasation into the perivascular space and tissue parenchyma in the following two days post-stroke [169],[170]. CD11b is also strongly expressed in the neutrophils of ischemic stroke patients as compared to healthy controls, reflecting the clinical severity of inflammatory response in the brain [163, 171].

Another protein expressed by PMNs in the adult rat brain is ANXA3. In the developing brain ANXA3 is actually produced by resting microglia (bioRxiv preprint doi: https://doi.org/10.1101/627539) as well as by activated microglia/macrophage cells in the infarcted area of young and aged rats [172]. Upregulation of ANXA3 is paralleled by an upregulation of Alox5 transcripts following cerebral ischemia [173]. Much like the fractalkine CX3CL1-CX3CR1 receptor that is present on injured but still viable neurons [118], neuronal 5-Lox mRNA expression strongly depends on the presence of microglia and might be an early response to neuroinflammation, strongly suggesting a feedback signal between neurons and microglia after brain injury.

Recent studies have also shown that after brain ischemia, microglia express the phagocytosis related proteins Milk fat globule EGF-like factor-8 (MFG-E8) and Mer receptor tyrosine kinase (MerTK) affecting still living and viable neurons at 3 to 7 days after focal brain ischemia [174-176].

*Slc6a20* mRNA is expressed by astrocytes and encodes an amino acid transporter that regulates proline and glycine levels and hence N-methyl-D-aspartate receptor (NMDAR) function in the mouse brain [177]. NMDAR is a glutamate receptor and ion channel that plays a central role in the CNS excitatory neurotransmission. Depending on its subunit composition, its ligands are glutamate and glycine. During brain development glycine (Gly) binds NMDAR and is involved in synapse removal by microglia acting in concert with astrocytes [177]. However, in the adult brain Gly-activation of NMDAR on microglia decreases dramatically along with decreased levels of the NR1 subunit paralleled by increases in the NR2 subunits [178].

*In vitro*, NMDAR stimulation of microglia induces their proliferation, morphological activation and release of pro-inflammatory mediators [179]. *In vivo*, NMDAR activation of microglia in the post-stroke adult brain causes an inflammatory response and triggers neocortical neuronal cell death. Furthermore, neuronal cell death was significantly reduced through pharmacological inhibition or genetically induced loss of function of the microglial NMDARs [180]. Following cerebral ischemia in mice, *Slc6a20* transcript has been reported to be downregulated while *Slc6a5* mRNA was upregulated [181].

Post-ischemic neuroinflammation also up-regulates *Litaf* transcripts in macrophages exposed to lipopolysaccharide (LPS) [117]. LITAF (Lipopolysaccharide-induced tumor necrosis factor alpha factor) plays a role in endosomal protein trafficking and in targeting proteins for lysosomal degradation. In addition, it regulates the expression of numerous cytokines, such as TNF, CCL2, CCL5, CXCL1, IL1A and IL10 [182].

Microglia processes constantly move in the area surrounding the cell body sensing any changes in the environment caused by cell death associated with neurodegeneration or injury-associated neuro-inflammation using cellular receptors and proteins collectively dubbed “sensome”. Such proteins include, among others, CD53, CD68, P2RY6, FCER1g, FCGR3, FCGR1, FCGR4, CSF1R [91]. Of these, CCSFR1, CD53, and FCGR3 are expressed both by microglia and macrophages [91].

The *Csf1* transcript encodes the colony stimulating factor 1 (CSF1), also known as macrophage colony-stimulating factor (M-CSF), a secreted cytokine which causes hematopoietic stem cells to differentiate into macrophages and that is essential for brain development [183]. More recently, CSF1 and its receptor CSFR1, showed significant upregulation in microglia surrounding the Abeta plaques and as such, has been proposed as a marker of neurodegenerative diseases [90]. Likewise, *Csf1* mRNA is increased in CSF1R-microglial-encephalopathy or glia-original dementia, a rare autosomal dominant disease caused by mutations in the gene coding for CSF1R resulting in microglial dysfunction [184]. Finally, *Csf1* transcripts were found to be upregulated in the ischemic hemisphere of a mouse model of stroke [86]. Further, the CSF1R inhibitor Ki20227, was neuroprotective after ischemia in mice most likely via inhibition of microglia M1 polarization and NLRP3 inflammasome pathway activation [185].

FCG gamma receptors (FCGRs) are also part of the microglial “sensome” that are upregulated in microglia following cerebral ischemia in animal models. FCGR plays a key role in macrophage activation. Indeed, in a mouse model of stroke Fcgr -/- mice showed significantly reduced mortality (20%) and smaller infarcts [186, 187].

*Mpeg1* mRNA encodes perforin-2 that is expressed by brain macrophages. Perforin-2/macrophage-expressed protein 1 is one of the oldest membrane attack complexes of complement [188]. In ischemic stroke the number of macrophages expressing perforin increase largely until day 3 after stroke, and then moderately decline [189].

The *Chi3l*1 mRNA encodes chitinase, an enzyme highly expressed along with GFAP and complement C3, in astrocytes during human brain development [15]. However, in the adult, post-ischemic brain *Chi3l1* mRNA is highly expressed by macrophages at day3 after cerebral ischemia [190].

*Arhgap*25 mRNA codes for ARHGAP25, a protein that is specifically expressed in macrophages to enhance their phagocytic activity [191] via modulation of the actin cytoskeleton [192]. In the brain, the *Arhgap*25 transcript has been associated with various processes like, inflammation and angiogenesis including cerebral cavernous malformations [193]. An increased expression of *Arhgap*25 mRNA has been recently reported in a rat model of cerebral ischemia [194].

**Figure 4.**
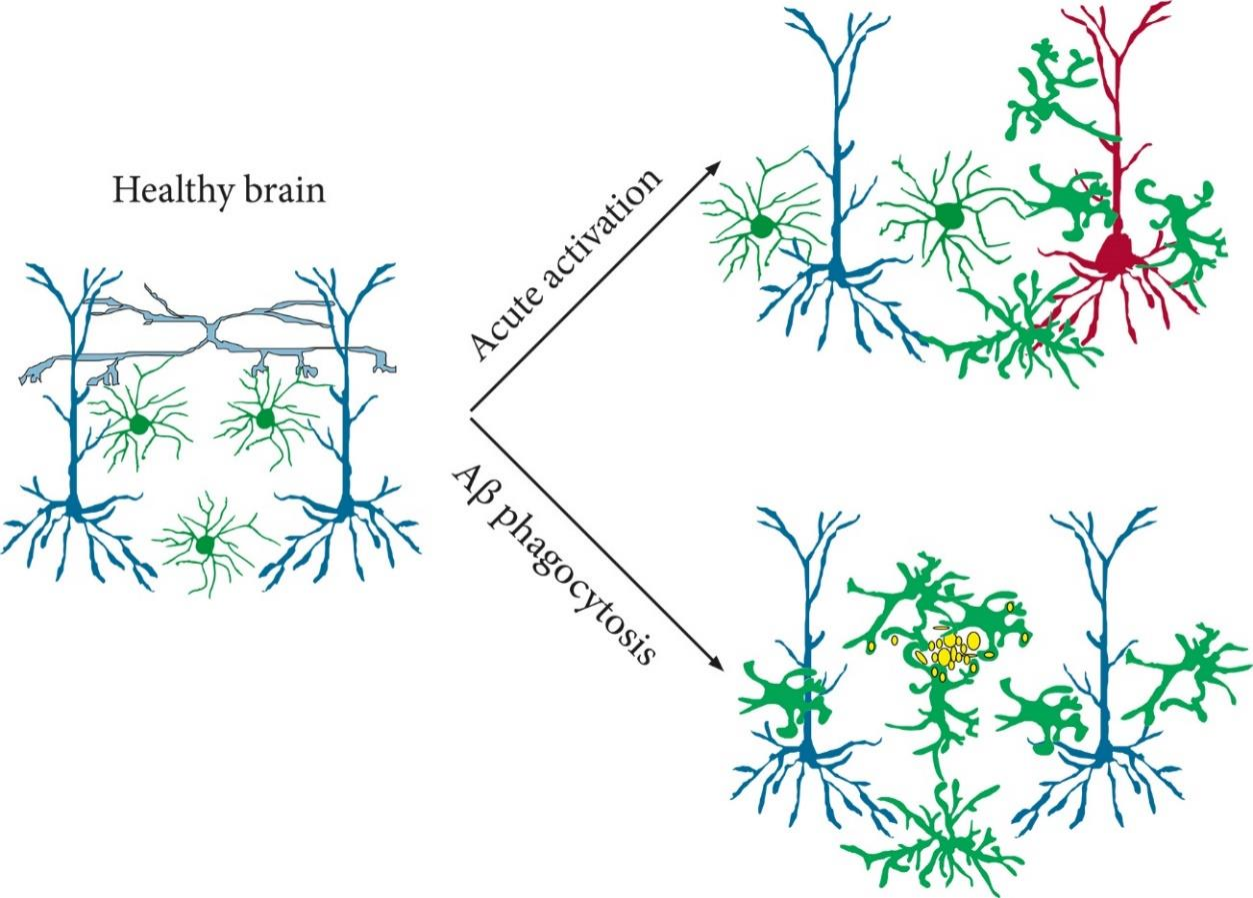
Cartoon depicting the role of microglia in the healthy and injured brain. Please note microglia proliferation and activation by beta amyloid deposits in the brains of Alzheimer’s disease patients and conversion to phagocytic macrophages following the acute phase injury.

The *Gpr34* mRNA encodes GPR34, a Gi/o protein-coupled receptor (GPCR) of the nucleotide receptor P2Y12-like group that is highly conserved among vertebrates. This receptor is highly expressed in microglia during brain development and is indispensable to microglia-dependent synaptic wiring via phagocytosis-mediated removal of synapses. Indeed, GPR34-deficient microglia showed reduced phagocytosis activity [92, 195]. In the adult brain, the GPCR family plays a pivotal role in the modulation of various components of microglial activation and migration to the damaged brain area [196]. Indeed, in a mouse model of ischemic stroke *Gpr34* mRNA was upregulated in macrophages at day3 following cerebral ischemia, suggesting a shift from the homeostatic function to phagocytic activity [190]. A cartoon depicting the role of microglia in the healthy and injured brain is shown in Figure 4.

### CONCLUSIONS

Although astrocytes and microglia are fundamentally different in origin and function, they often affect the same developmental processes such as neuro-/gliogenesis, angiogenesis, axonal outgrowth, synaptogenesis, and synaptic pruning. In the adult brain astrocytes are a critical player in the synapse remodeling by mediating synapse elimination while microglia activity has been associated with changes in synaptic plasticity, showing a decrease in the motility of the microglial processes during low neuronal activity and constantly sensing the environment and remove cell debris. However, in the lesioned brain astrocytes proliferate and play essential functions with regard to energy supply to the neurons, neurotransmission and buildup of a protective scar isolating the lesion site from the surroundings. Inflammation, neurodegeneration, or loss of brain homeostasis induce changes in gene expression and microglial morphology and function, generally referred to as “primed” microglia. This change in gene expression is characterized by an enrichment of phagosome, lysosome, and antigen presentation signaling pathways and is associated with an up-regulation of genes encoding cell surface receptors. In addition, primed microglia are characterized by upregulation of a network of genes in response to interferon gamma. A better understanding of the origin, differentiation process and developmental functions of microglia and astrocytes will help us to better understand their role both in the developing as well as the adult brain that in turn may lead to new therapeutic strategies with which to protect the aging brain and improve neurorestoration in neurovascular diseases.
